# MS2-CL: Multi-Scale Self-Supervised Learning for Camera to LiDAR Cross-Modal Place Recognition

**DOI:** 10.3390/s26051561

**Published:** 2026-03-02

**Authors:** Wen Liu, Lei Ma, Xuanshun Zhuang, Zhongliang Deng

**Affiliations:** School of Electronic Engineering, Beijing University of Posts and Telecommunications, Beijing 100876, China; malei@bupt.edu.cn (L.M.); zxs2024@bupt.edu.cn (X.Z.);

**Keywords:** cross-modal place recognition, self-supervised learning, Swin Transformer, autonomous driving

## Abstract

Place recognition is a fundamental challenge for robotics and autonomous vehicles. While visual place recognition has achieved high precision, cross-modal place recognition—specifically, visual localization within large-scale point cloud maps—remains a formidable problem. Existing methods often struggle with the significant domain gap between modalities and can be computationally prohibitive, especially those processing raw 3D point clouds. Furthermore, they frequently fail to learn features invariant to viewpoint and scale variations, limiting generalization to unseen environments. In this paper, we formulate cross-modal recognition as a problem of learning a scale-invariant, unified embedding space. Our framework employs a hierarchical Swin Transformer to extract multi-scale features from unified 2D representations of both modalities. The central principle of our method is a multi-scale self-distillation paradigm, which recasts feature learning as an intra-modal knowledge transfer task. Specifically, the coarse-scale “teacher” features provide supervision for the fine-scale “student” features. The final inter-modal alignment is then achieved via a global contrastive loss, exclusively leveraging the semantically rich “teacher” embeddings to ensure a reliable and discriminative matching. Extensive experiments on the KITTI and KITTI-360 datasets demonstrate that our method achieves state-of-the-art performance. Notably, using only the KITTI-trained model without fine-tuning, Recall@1 exceeds 60% on all evaluable sequences of KITTI-360 at a 10 m threshold. Code and pre-trained models will be made publicly available upon acceptance.

## 1. Introduction

Visual place recognition plays a crucial role in mobile robotics, particularly in applications such as autonomous driving and simultaneous localization and mapping (SLAM). It relies on sensor data, such as images and point clouds, to achieve global localization and loop closure detection in pre-built maps and databases. Visual place recognition [1,2,3] is highly competitive due to the small size and low cost of cameras. However, cameras are sensitive to changes in lighting and field of view. Lidar, on the other hand, provides precise geometric information and is robust to illumination changes, making it the standard for building High-Definition (HD) maps.

In practical autonomous driving scenarios, a major challenge is balancing system performance with hardware costs. While mapping vehicles are typically equipped with high-end LiDARs to construct accurate 3D HD maps, mass-produced passenger vehicles often rely on low-cost cameras for perception. Therefore, the ability to localize a camera-equipped vehicle within a pre-built LiDAR map is of significant practical value. This task, known as cross-modal place recognition, enables a cost-effective localization solution that leverages the structural accuracy of LiDAR maps without imposing heavy hardware costs on end-user vehicles. Given the discrepancies between the data modalities queried by mobile robots and those stored in the database, cross-modal place recognition tackles the challenges arising from these modality differences, thereby significantly enhancing the accuracy and resilience of global place recognition.

**Figure 1:**
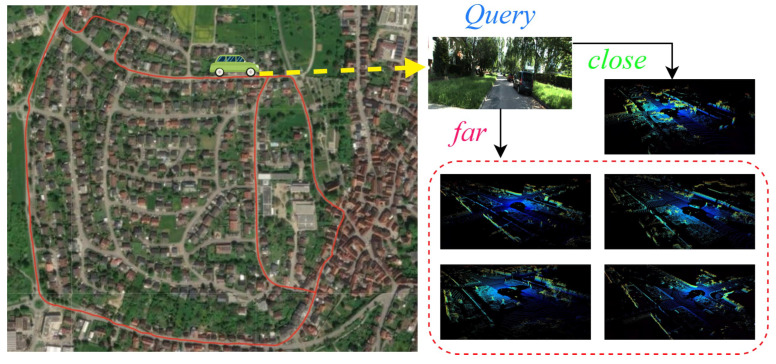
The objective of MS2-CL is to perform visual localization within large-scale point cloud maps, specifically to retrieve the most similar point cloud corresponding to a given query image.

For example, an autonomous vehicle can achieve precise visual place recognition on a pre-built large-scale 3D point cloud map using query images captured by an inexpensive camera, as shown in Figure 1. Given the complementary nature of image and point cloud data, several multimodal fusion methods [4,5,6] have been developed to combine 2D and 3D data, significantly improving the precision of global localization. However, due to the inherent differences between these two modalities, fusion remains a challenging task. Furthermore, existing multimodal methods have not fully tackled the issue of performing global place recognition from one modality’s sensor to another modality’s map. To overcome the data representation differences between image and point cloud information, some methods [7,8] convert sensor data into intermediate representations, such as bird’s-eye views or depth images. Others [9,10,11] aim to extract structural information from visual data to align it with point clouds or directly use deep neural networks to match the data. These methods, however, are resource-intensive and involve large model parameters. Specifically, processing 3D point clouds often requires handling vast and complex data, which leads to high hardware costs. Moreover, even with sufficient training data, models may struggle to maintain strong performance on unseen data.

To address these limitations, we propose a novel and efficient framework for cross-modal place recognition, designed to learn discriminative, scale-invariant features while minimizing computational overhead. The core idea is to bridge the modality gap by first transforming both sensor inputs into a unified 2D representation and then leveraging a multi-scale self-distillation paradigm within a Transformer-based architecture. We first project the 3D point cloud into a 2D spherical representation and estimate a dense depth map from the query RGB image. This step not only creates a common ground for feature extraction but also significantly reduces the complexity associated with processing raw 3D data. Subsequently, we employ a shared-weight Swin Transformer backbone to extract hierarchical features from both 2D representations. The strength of the Swin Transformer lies in its ability to capture both local details and global context through its shifted window mechanism, producing feature maps at multiple scales. The key innovation of our work lies in how we supervise the learning of these multi-scale features. We introduce a multi-scale teacher–student learning strategy, a form of self-distillation. Specifically, we designate the features from the coarsest scale (e.g., the final stage), which encapsulate the most abstract and global semantic information, as the “teacher”. The features from finer scales (e.g., the earlier stages), which retain more spatial details, act as “students”. Through a Scale Consistency Supervision loss, we compel the student features to align with the teacher’s representation. This process enforces intra-modal consistency, forcing the network to learn features that are invariant to scale variations and viewpoint changes.

Finally, the global descriptors extracted from the “teacher” level of both modalities are used to compute a Global Contrastive Loss. Inspired by the success of contrastive learning paradigms like Contrastive Language-Image Pre-training (CLIP) [12] in pairing heterogeneous data, and in contrast to methods relying on triplet loss, this loss function pushes the descriptors of corresponding image-point cloud pairs closer together in the embedding space while pulling all other non-matching pairs within the batch apart. By integrating intra-modal scale consistency with inter-modal contrastive learning, our model effectively learns a unified embedding space that is highly discriminative.

In summary, the main contributions of our work are as follows:

- We propose a lightweight framework that unifies 2D image and 3D point cloud into common 2D representations, enabling the use of an efficient Swin Transformer backbone for cross-modal place recognition.
- We introduce a novel multi-scale teacher–student self-distillation paradigm. By enforcing consistency between coarse-scale (teacher) and fine-scale (student) features within each modality, our model learns highly discriminative and scale-invariant descriptors.
- Our method achieves state-of-the-art performance on the KITTI and KITTI-360 benchmarks. Notably, a model trained solely on the KITTI dataset demonstrates exceptional generalization, achieving a Recall@1 exceeding over 57.5% on eight sequences from KITTI-360 at 20 m threshold, without any fine-tuning.

## 2. Related Work

### 2.1. Image-Based Place Recognition

Early visual place recognition research relied on manually designed features like SIFT [13] and ORB [14], which are designed to extract local features from the image. These methods performed well in static environments but were sensitive to lighting changes. With the advent of deep learning, CNN-based Place Recognition techniques [15] have seen substantial advancements. NetVLAD [1] introduced weakly supervised learning via triplet loss, enhancing feature associations. Integrating generalized mean pooling (GeM) [16] with contrastive learning significantly enhances the discriminative power of the extracted features. Hybrid architectures like Patch-NetVLAD [17] optimized local and global features through hierarchical fusion. Several researchers [2] have leveraged the Transformer architecture to tackle the challenge of image geolocation from multiple angles, effectively addressing the limitations inherent in traditional methods based on convolutional neural networks. Recently [18], depth estimation has provided geometric verification for feature matching, and a two-stage retrieval process (global coarse localization followed by local fine matching) has markedly improved search efficiency in large-scale scenes.

### 2.2. Point-Cloud-Based Place Recognition

In recent years, point cloud-based place recognition methods have advanced along two primary directions: direct point cloud processing and structured projection. PointNetVLAD [19] integrates deep learning by generating descriptors via PointNet [20] and NetVLAD [1], but its point-wise mechanism limits local geometric modeling. Structural projection methods, including distance image-based projection [21,22] and bird’s-eye view-based projection [23,24], transform unordered point cloud data into two-dimensional representation formats. OverlapNet [21] estimates the overlap and relative pose between 3D point clouds by leveraging siamese networks and channel attention mechanisms. BVMatch [23] generates descriptors by fusing multi-scale bird’s-eye view (BEV) features and employs the Bag-of-Words (BoW) method for frame retrieval. Recently, researchers [25] have improved robustness to field-of-view changes by fusing range and BEV images using cross-view transformers, achieving reliable place recognition.

### 2.3. Image to Point Cloud Place Recognition

Currently, cross-modal place recognition from images to point clouds remains imprecise and low accuracy despite significant advancements in single-modal place recognition. Consequently, there is considerable potential for further exploration in achieving image-based place recognition on large-scale point cloud maps. Previous researchs [26,27] have primarily focused on extracting shared embedding representations between 2D images and 3D point clouds but has largely overlooked the alignment and interaction of information across these two modalities, leading to suboptimal generalization performance. Methods such as those described in [28,29] focus on detecting local features within images and point clouds, facilitating the identification of cross-modal correspondences for place retrieval. The 2D3D-MatchNet framework [29] generated descriptors for images and point clouds using VGG and PointNet networks, respectively, and was trained using triplet loss. However, this approach is limited to small-scale environments due to the computational demands of dense point cloud representation, making it challenging to scale effectively to larger environments. (LC)^2^ [8] integrates RGB images and point clouds into a unified 2D depth images, encoding them into vetor representations, and subsequently optimizing these representations via contrastive and triplet losses through a two-stage network training. I2P-Rec [7] leverages a depth estimation algorithm to reconstruct point clouds from images, mitigating modality differences, and utilizes BEV projection for global feature extraction to accurately identify and locate camera images within large-scale point cloud maps. LIP-Loc [30] achieves cross-modal Place Recognition by leveraging batch contrastive loss for pre-training with a large number of image and point cloud pairs. ModaLink [31] introduces a field-of-view transformation module that converts point clouds into range images, aligning modality information and providing more discriminative global descriptors through its designed encoder. However, these methods still fall short of achieving superior recognition accuracy at high precision levels.

## 3. Methods

This paper introduces MS2-CL, a lightweight cross-modal place recognition framework capable of achieving high accuracy on both seen and unseen datasets.

### 3.1. Data Processing

(1) *Range image generation:* The point cloud data, represented in sparse Cartesian coordinates (x,y,z), is transformed into a 2D range image to unify the modalities by emulating the spherical projection characteristics of a LiDAR sensor. Following an initial filtering stage that removes points exceeding the predefined maximum detection range $R_{max}$, we calculate the Euclidean distance from each remaining point to the sensor origin as the depth measurement $r_{i}$.

(1) $$P=\{p_{i}|p_{i}={\left(x_{i},y_{i},z_{i}\right)}_{i=1}^{N}\},$$

(2) $$P^{\prime }=\{p_{i}|0<∥v_{i}{∥}_{2}<R_{max},v_{i}=\left(x_{i},y_{i},z_{i}\right)\},$$

(3) $$r_{i}=\sqrt{x_{i}^{2}+y_{i}^{2}+z_{i}^{2}},$$

For the remaining valid points, we calculate their horizontal (yaw $\varphi_{i}$) and vertical (pitch $\theta_{i}$) angles based on their 3D coordinates.

(4) $$\varphi_{i}=-\arctan\left(\frac{y_{i}}{x_{i}}\right),$$

(5) $$\theta_{i}=\arcsin\left(\frac{z_{i}}{r_{i}}\right),$$

These angles are then used to determine the corresponding positions in the 2D projection plane. Specifically, the horizontal angle is mapped to a normalized interval, while the vertical position is computed proportionally to the total field of view. The resulting coordinates are scaled to fit the preset image dimensions, ensuring accurate placement of each point in the range image R(u,v).

(6) $$u_{i}=\left(\frac{\theta_{i}}{\pi }+1\right)\cdot \frac{W}{2},$$

(7) $$v_{i}=\left(1-\frac{|\varphi_{i}\left|+\right|\alpha_{down}|}{\alpha }\right)\cdot H,$$

where $\alpha =|\alpha_{down}\left|+\right|\alpha_{up}|$ is the total vertical field of view and *H* and *W* represent the height and width of the range image, respectively. It is important to note that the spherical projection parameters, specifically the vertical field of view angles $\alpha_{up}$ and $\alpha_{down}$, are sensor-dependent. In this work, these parameters are configured to match the Velodyne HDL-64E LiDAR sensor used in the KITTI datasets. For deployment on vehicles with different LiDAR configurations (e.g., 32-beam or solid-state LiDARs), these projection parameters must be adjusted to align with the specific sensor’s vertical field of view to ensure optimal image-to-point cloud alignment. To address potential occlusions due to overlapping points in the original point cloud, we sort all valid points by their distance values in ascending order, ensuring that closer points are not obscured by farther ones. Finally, a range image matrix is constructed, where each element represents the depth value at the corresponding pixel location. Additionally, for each pixel of the generated range image, which represents depth information, multiply by a fixed scaling factor β to enhance the contrast of the whole image.

(8) $$R^{\prime }\left(u,v\right)=\beta *R\left(u,v\right),$$

(2) *Deep Image Generation:* This study utilizes the Monodepth2 [32] framework to process monocular RGB image sequences from the KITTI and KITTI-360 datasets. We selected this method due to its temporal self-supervised learning approach based on monocular video, which constructs a differentiable photometric reprojection loss using camera motion parameters between consecutive frames. This approach implicitly decouples scene depth from motion estimation, effectively mitigating the monocular scale ambiguity issue. To further enhance robustness in dynamic traffic scenes, the method incorporates a multi-frame consistency-driven adaptive masking mechanism [33]. This mechanism effectively filters out moving objects (e.g., other vehicles) that violate the static scene assumption, significantly reducing reprojection error noise in dynamic regions. In this work, RGB images are processed offline at their original resolution, and the resulting depth images from monocular estimation are stored locally, substantially decreasing the time and computational resources required for the entire training process.

### 3.2. Multi-Scale Self-Supervised Learning Network

(1) *Network Architecture:* Our proposed framework for cross-modal place recognition employs a dual-stream, multi-scale architecture to achieve coherent feature alignment between visual and LiDAR data. This design comprises three core stages: modality-specific preprocessing, hierarchical feature encoding under a teacher–student paradigm, and a dual-objective loss function for comprehensive supervision. The network’s backbone consists of parallel Swin Transformer encoders, each followed by dedicated projection heads.

**Figure 2:**
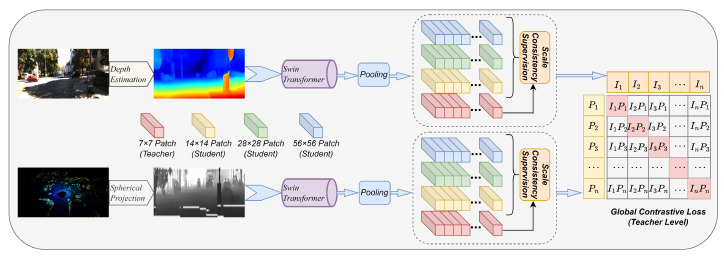
The architecture of the proposed multi-scale self-supervised learning network.

As depicted in Figure 2, the framework begins with modality-specific preprocessing. The camera stream converts an RGB image into a dense depth map, while the LiDAR stream transforms a 3D point cloud into a 2D spherical projection. These 2D representations are then fed into their respective Swin Transformer backbones, which extract features at multiple hierarchical scales. A key aspect of our architecture is the intra-modal teacher–student learning paradigm. For each modality, features from the encoder’s final, most global stage (e.g., 7 × 7 patch level) are designated as the ‘teacher’ embedding, representing a holistic scene view. Features from earlier, higher-resolution stages are termed ‘student’ embeddings. This structure facilitates a dual-objective optimization. First, an intra-modal Scale Consistency Supervision loss aligns the student embeddings with their corresponding teacher embedding, ensuring feature consistency across granularities. Second, the main Global Contrastive Loss is computed exclusively between the teacher-level embeddings of the two modalities. This primary loss drives the network to map corresponding camera and LiDAR views to a unified embedding space. This combined supervision enables the learning of discriminative descriptors for high-precision cross-modal place recognition.

(2) *Scale Consistency Supervised Learning:* Distinction from Conventional Self-Distillation: Our scale consistency supervision differs fundamentally from conventional knowledge distillation frameworks. Traditional self-distillation methods (e.g., [34]) typically distill from a larger teacher model to a smaller student for model compression. In contrast, we perform *intra-modal*, *multi-scale self-distillation* within a single encoder, where teacher and student roles are assigned based on *feature granularity* rather than model size. Specifically, the coarsest-scale embedding (capturing global semantics) supervises finer-scale embeddings (retaining spatial details), enforcing hierarchical consistency across scales. This design is tailored for cross-modal alignment: by ensuring scale-invariant representations within each modality *before* cross-modal matching, we effectively bridge the camera-LiDAR domain gap. The stop-gradient operation on teacher features prevents interference with the primary contrastive loss, ensuring stable optimization. Unlike existing methods that focus on inter-modal alignment alone, our intra-modal scale consistency provides a crucial regularization that improves feature robustness to viewpoint and scale variations.

Beyond the primary cross-modal alignment, we introduce an auxiliary self-supervised task to enforce feature consistency across different scales within each modality. This is achieved through an intra-modal teacher–student learning paradigm. For a given modality (e.g., camera depth images), we designate the embedding from the final, most global encoder stage as the ‘teacher’ embedding, $z_{D}^{T}$, which captures a coarse-grained, holistic representation of the scene. Embeddings from the preceding, higher-resolution stages, ${\left\{z_{D}^{s}\right\}}_{s=1}^{S-1}$, are treated as ‘student’ embeddings, representing finer-grained local features.

The Scale Consistency Loss, $L_{SC}$, then minimizes the distance between each student embedding and its corresponding teacher embedding. Crucially, the teacher embedding is treated as a fixed target by stopping the gradient flow from this auxiliary loss, ensuring that its representation is guided solely by the main global contrastive task. This process is applied symmetrically to both the camera depth (D) and LiDAR range (R) images. The loss for each modality is formulated as:

(9) $$\begin{array}{cc} L_{SC}^{D} & =\frac{1}{S-1}\sum_{s=1}^{S-1}L_{dist}\left(z_{s}^{D},\mathrm{sg}\left(z_{T}^{D}\right)\right), \end{array}$$

(10) $$\begin{array}{cc} L_{SC}^{R} & =\frac{1}{S-1}\sum_{s=1}^{S-1}L_{dist}\left(z_{s}^{R},\mathrm{sg}\left(z_{T}^{R}\right)\right), \end{array}$$

where *S* is the total number of scales, sg(·) denotes the stop-gradient operation, and $L_{dist}$ is a distance metric, for which we use the Smooth L1 loss. The total objective for scale consistency supervision is the sum of the individual modality losses:

(11) $$L_{SC}=L_{SC}^{D}+L_{SC}^{R}.$$

This auxiliary objective encourages the model to learn multi-scale descriptors that are hierarchically consistent, thereby enhancing feature robustness against variations in viewpoint and distance.

(3) *Global Contrastive Loss Function:* Different from the triplet loss commonly employed in previous methods, we train the proposed network architecture for Image-to-Point Cloud correspondence retrieval using a cross-modal mini-batch contrastive loss function. Given a batch of 2D depth image descriptors ${\left\{E_{i}^{D}\right\}}_{i=1}^{N}$ and their corresponding matching 2D range image descriptors ${\left\{E_{i}^{R}\right\}}_{i=1}^{N}$, where *N* represents the size of mini-batch, this batch contains *N* positive pairings and $N^{2}-N$ negative pairings. For each pairing, the contrastive loss is computed as follows.

(12) $$\begin{array}{cc} l(i,D,R) & =-\log\frac{\exp(E_{i}^{D}\cdot E_{i}^{R}/\tau )}{\sum_{j\in N}\exp\left(E_{i}^{D}\cdot E_{j}^{R}/\tau \right)}\\ \; & \;-\log\frac{\exp(E_{i}^{R}\cdot E_{i}^{D}/\tau )}{\sum_{j\in N}\exp\left(E_{i}^{R}\cdot E_{j}^{D}/\tau \right)}, \end{array}$$

where τ is the temperature, similar to CLIP [12]. The parameter τ scales the logits to control the sharpness of the probability distribution. A properly chosen τ assists the model in mining hard negatives by emphasizing difficult samples during gradient computation. Following standard practices [30], we empirically set τ=1.0 in our experiments. Throughout the training process using mini-batch, the objective function for the paired contrastive loss between images and point clouds is defined as follows:

(13) $$L\left(D,R\right)=\frac{1}{N}\left[\sum_{i\in N}l\left(i,D,R\right)\right].$$

(4) *Training Strategy:* Our training process, detailed in Algorithm 1, is driven by the joint optimization of the global contrastive loss and the intra-modal scale consistency loss in a self-supervised manner. We employ the AdamW optimizer for model training and adopt a differential learning rate strategy. Specifically, the learning rates for the depth and range image encoders are set to $1\times 10^{-4}$, while the projection heads are trained with a higher learning rate of $1\times 10^{-3}$ to facilitate faster adaptation. The weighting factor λ for the scale consistency loss is set to 0.5. To ensure training stability, gradients are clipped with a maximum norm of 1.0.
**Algorithm 1** Multi-Scale Self-Supervised Cross-Modal Learning (MS2-CL)**Require:** Depth image $I^{cam}$, LiDAR range image $I^{lid}$, temperature τ, loss weight λ**Ensure:** Cross-modal matching model with scale-invariant embeddings  1:**Initialize:**  2:    Multi-scale Swin encoders $E_{cam},E_{lid}$  3:    Projection heads ${\left\{P_{cam}^{s}\right\}}_{s=1}^{4},{\left\{P_{lid}^{s}\right\}}_{s=1}^{4}$  4:    Scale consistency loss $L_{sc}$ and contrastive loss $L_{ctr}$  5:   6:**Step 1: Multi-scale feature extraction**  7:${\left\{F_{cam}^{s}\right\}}_{s=1}^{4}\leftarrow E_{cam}\left(I^{cam}\right)$  8:${\left\{F_{lid}^{s}\right\}}_{s=1}^{4}\leftarrow E_{lid}\left(I^{lid}\right)$  9: 10:**Step 2: Feature projection at each scale**11:**for** s=1 **to** 4 **do**12:    $Z_{cam}^{s}\leftarrow P_{cam}^{s}\left(F_{cam}^{s}\right)$13:    $Z_{lid}^{s}\leftarrow P_{lid}^{s}\left(F_{lid}^{s}\right)$14:**end for**15: 16:**Step 3: Teacher–student separation (coarse → teacher, fine → student)**17:$Z_{cam}^{T}\leftarrow Z_{cam}^{4}$,   $Z_{lid}^{T}\leftarrow Z_{lid}^{4}$18:$S_{cam}\leftarrow \left\{Z_{cam}^{1},Z_{cam}^{2},Z_{cam}^{3}\right\}$19:$S_{lid}\leftarrow \left\{Z_{lid}^{1},Z_{lid}^{2},Z_{lid}^{3}\right\}$20: 21:**Step 4: Intra-modal scale consistency supervision**22:$L_{sc}^{cam}\leftarrow \frac{1}{3}\sum_{Z\in S_{cam}}\;\;{∥Z-Z_{cam}^{T}∥}_{1}$23:$L_{sc}^{lid}\leftarrow \frac{1}{3}\sum_{Z\in S_{lid}}\;\;{∥Z-Z_{lid}^{T}∥}_{1}$24: 25:**Step 5: Cross-modal contrastive learning (teacher embeddings only)**26:Compute logits: $\ell_{ij}=\frac{Z_{lid,i}^{T}\cdot Z_{cam,j}^{T}}{\tau }$27:$L_{ctr}\leftarrow \frac{1}{2}\left(\mathrm{CE}\left(\ell \right)+\mathrm{CE}\left(\ell^{⊤}\right)\right)$28: 29:**Step 6: Final objective**30:$L=L_{ctr}+\lambda \left(L_{sc}^{cam}+L_{sc}^{lid}\right)$31:**return** Trained model capable of cross-modal place recognition.

## 4. Experiments

In this section, we evaluate the performance of our MS2-CL method in image-based place recognition on large-scale point cloud maps. Using the KITTI and KITTI-360 datasets, we assess the recall@N(%) accuracy of MS2-CL and compare it against several baseline models. Furthermore, we perform a zero-shot transfer analysis of our method on the KITTI-360 dataset. Lastly, ablation studies are conducted to investigate the contributions of key algorithms in our approach.

### 4.1. Dataset

**KITTI.** Evaluation is conducted on the KITTI odometry dataset, which provides a large number of synchronized and calibrated LiDAR point clouds and RGB images. This dataset is widely adopted for validating autonomous driving–related algorithms. KITTI consists of 22 sequences (00–21). In our experimental setup, we define sequences **02, 05, 06, and 08 as the testing set** to evaluate generalization performance. All remaining sequences (00, 01, 03, 04, 07, 09–21) constitute the training set. Note that while the official odometry benchmark does not provide ground truth for sequences 11–21, we obtain their precise poses from the raw GPS/IMU data included in the KITTI raw dataset, following the practice in LIP-Loc [30]. Additionally, we report the results on Sequence 00 in Table 1 to demonstrate the model’s fitting capability on the training data.

**KITTI-360.** To further investigate the zero-shot transferability of our method, we utilize the KITTI-360 dataset. It comprises a Velodyne HDL-64E LiDAR (Velodyne Lidar, San Jose, CA, USA)sensor and raw images captured by the perspective camera. This dataset contains approximately 80,000 frames of lidar and image pairs covering a distance of 73.7 km, along with the precise vehicle pose information required for evaluation. We selected sequences 3, 4, 5, 6, 7, 9 and 10 for training and cross-validation and performed the evaluation using sequence 0.

### 4.2. Implementation and Experimental Setup

Our MS2-CL utilizes a pretrained swin-tiny-patch4-window7-224 as the backbone. For range image generation, we set the detection distance to 50 m, the upward field of view ($\alpha_{up}$) to 3 degrees, and the downward field of view ($\alpha_{down}$) to −25 degrees. The range image size is configured to be 64 × 900 pixels. Prior to feeding into the encoder, all input images are uniformly resized to 224 × 224 pixels.

### 4.3. Evaluation Metrics

(1) On the KITTI dataset, we evaluate our model using recall rates at Top-1 and Top-1%. A match between a query image and a retrieved point cloud is deemed positive if their Euclidean distance is less than 10 m. Recall at Top-N, a standard metric for retrieval tasks, is defined as the percentage of queries for which at least one correct match is found within the top-N ranked candidates. It is formally calculated as:

(14) $$\mathrm{Recall}@N=\frac{1}{\left|Q\right|}\sum_{q\in Q}I\left[R_{N}\left(q\right)\cap D_{q}^{+}\neq Ø\right],$$

where *Q* is the set of all queries, $R_{N}\left(q\right)$ is the set of top-N retrieved candidates for a given query *q*, and $D_{q}^{+}$ is the set of all ground truth positive matches for *q* in the database. The indicator function I[·] returns 1 if its condition is true (i.e., at least one positive match exists in the top-N results) and 0 otherwise.

**Recall@Top-1% Definition:** The Recall@Top-1% metric extends this concept by evaluating retrieval performance within the top 1% of the database. Formally:

(15) $$\mathrm{Recall}@\mathrm{Top}\text{-}1\%=\frac{1}{\left|Q\right|}\sum_{q\in Q}I\left[R_{\left\lceil 0.01\times |D|\right\rceil }\left(q\right)\cap D_{q}^{+}\neq Ø\right]$$

where |D| is the database size (number of point clouds) for the sequence, ⌈0.01×|D|⌉ denotes the top 1% of candidates (rounded up), $R_{k}\left(q\right)$ represents the top-*k* retrieved point clouds for query *q*, and $D_{q}^{+}$ denotes the set of positive (correct) matches for query *q*. For example, in a sequence with 4500 point clouds in the database, Top-1% evaluates whether a correct match appears within the top 45 (⌈4500×0.01⌉=45) retrieved candidates. This metric assesses retrieval quality beyond the single best match, which is critical for downstream re-ranking or geometric verification stages in practical SLAM systems. This metric has been widely adopted in prior place recognition literature [20,23,33].

Specifically, for each test sequence, the **Query Set** *Q* is constructed using all RGB images in that sequence, while the **Database Set** comprises all corresponding LiDAR point clouds. Consequently, for a sequence of length *M*, both the query and database sets contain *M* samples. The retrieval is performed within the same sequence, where each query image searches against the entire point cloud database of that sequence.

(2) On the KITTI-360 dataset, we utilize recall rates at TOP-1, TOP-5, and TOP-20 as evaluation metrics. A match is deemed positive if the Euclidean distance between the query image and the retrieved point cloud is less than 20 m.

### 4.4. Evaluation for Place Recognition on the KITTI Dataset

We compare our method with baseline [28], I2P-Rec [7], (LC)^2^ [8], LIP-Loc [30] and ModaLink [31] on the KITTI dataset. The baseline approaches employs DNN to process images and point clouds, generating descriptors using NetVLAD [1]. I2P-Rec utilizes various depth estimation algorithms such as monocular depth estimation (MIM [35]), stereo matching estimation (PSM [36] and LEA [37]) to recover point clouds. It then generates descriptors based on BEV images of the recovered point clouds and uses PointNetVLAD to generate global descriptors for the point clouds. LC^2^ [8] transforms RGB images and point clouds into 2.5D depth images and performs a two-stage training process utilizing distinct loss functions. LIP-Loc [30] leverages large-scale pre-trained image-point cloud pairs and uses a batch contrastive loss function to bring them closer in the embedding space. ModaLink [31] transforms point clouds into image perspectives and relies on explicit FoV transformations for alignment, utilizing CNNs and NMF with Triplet Loss to generate descriptors. Distinct from ModaLink’s reliance on rigid geometric alignment, our proposed MS2-CL introduces a Teacher–Student Scale Consistency strategy within a Swin Transformer architecture. This enables implicit feature-level learning and utilizes a global Contrastive Loss, ensuring superior robustness compared to the local constraints of Triplet Loss.

**Monocular vs. Stereo Input Analysis:** It is noteworthy that our MS2-CL achieves state-of-the-art performance using only *monocular depth estimation*, while some strong baselines (PSM-I2P-Rec*, LEA-I2P-Rec*) require *stereo camera systems*. As shown in Table 2, our method achieves +13.1% higher average Recall@1 than stereo-based LEA-I2P-Rec* while requiring only half the cameras:

This demonstrates that our multi-scale contrastive learning framework can extract more discriminative cross-modal features even from limited visual input. The monocular design also offers significant practical advantages: (1) *Lower cost:* single-camera systems are significantly cheaper than stereo rigs; (2) *Reduced complexity:* no camera synchronization or baseline calibration required; (3) *Better robustness:* no failure modes from stereo matching errors; (4) *Easier deployment:* compatible with existing monocular camera infrastructure in production vehicles.

As shown in Table 1, our proposed MS2-CL, which relies solely on monocular images, significantly outperforms I2P-Rec across all scenarios involving depth estimation or panoramic images, and surpasses ModaLink trained with triplet loss in all sequences.

Furthermore, to demonstrate the remarkable performance of our method in cross-modal place recognition, we adopted progressively stringent evaluation criteria, as illustrated in Table 3. Specifically, we utilized a **Top-1** recall rate and set distance thresholds at 1 m, 4 m, 7 m, 10 m, 13 m and 16 m. To rigorously benchmark our performance, we compared MS2-CL with the state-of-the-art baseline ModaLink [31] under identical settings. As evidenced in Table 3, MS2-CL exhibits superior robustness, particularly on the challenging KITTI-02 sequence where it surpasses the baseline by **8.8%** at the strictest 1m threshold. Although ModaLink shows competitive results on sequence 05 at relaxed thresholds, our method consistently secures the highest recall at the precise 1 m threshold across all evaluated sequences. Overall, the experimental results confirm that MS2-CL consistently achieves an accuracy rate exceeding **68%** even under the tightest geometric constraints.

### 4.5. Evaluation for Place Recognition on the KITTI-360 Dataset

We compare our method with AECMLoc [38], a technique that relies on fisheye images and necessitates more intricate preprocessing, as well as the benchmark LIP-Loc [30] for cross-modal localization on the KITTI-360 dataset. Zero-shot LIP-Loc was trained using all sequences selected from the KITTI dataset. To ensure a fair comparison, our Zero-shot MS2-CL model was trained on the exact same set of sequences.

As illustrated in Table 4, based on the LIP-Loc evaluation metrics, our method has achieved an accuracy rate approaching **79.8%**, and the initial evaluation results provide evidence of zero-shot MS2-CL strong generalization capability.

**Figure 3:**
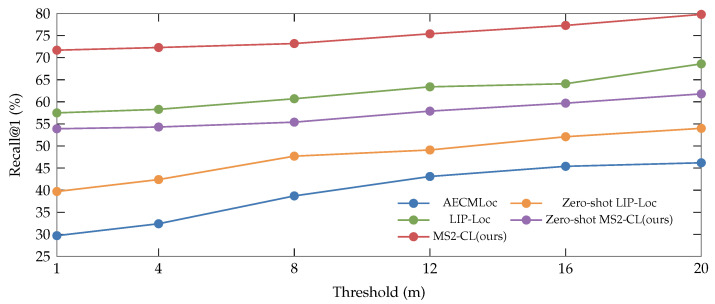
Evaluation of localization tolerance on the KITTI-360 dataset.

Moreover, our proposed method MS2-CL, as shown in Figure 3, is benchmarked against state-of-the-art methods including LIP-Loc and AECMLoc. The results demonstrate that MS2-CL consistently outperforms all baselines across the entire range of thresholds.

### 4.6. Zero-Shot Generalization Analysis

To rigorously evaluate the generalization capability of our MS2-CL model, we performed a challenging zero-shot cross-dataset evaluation. The model, trained exclusively on the KITTI dataset, was directly tested on the KITTI-360 dataset without any fine-tuning or retraining. As highlighted by prior work [38], this task presents a significant domain shift due to fundamental differences in sensor hardware and data acquisition: KITTI-360 utilizes a 180° fisheye camera, whereas KITTI employs a 90° standard perspective camera. This leads to drastic variations in image distortion and field of view. Furthermore, the two datasets exhibit no trajectory overlap, ensuring that the model is evaluated on entirely new environments. These factors collectively establish KITTI-360 as a true out-of-distribution testbed for our KITTI-trained model.

The quantitative results of this zero-shot evaluation are presented in Table 5. Despite the substantial domain gap and the absence of any adaptation, MS2-CL demonstrates remarkable generalization performance. The model achieves an average Recall@1 of **61.4%** across all eight test sequences.

### 4.7. Ablation Study

To validate the effectiveness of our proposed Scale Consistency Loss and to determine its optimal contribution to the overall training objective, we conducted a series of ablation studies on the KITTI dataset. The results, summarized in Table 6, analyze the model’s performance by varying the loss weight λ.

**Effectiveness of Scale Consistency Loss:** The most significant finding is the critical role of the Scale Consistency Loss ($L_{SC}$). By setting λ=0, we establish a baseline model trained solely with the global contrastive loss. Compared to our final model (λ=0.5), this baseline exhibits a catastrophic drop in performance. Specifically, Recall@1 plummets by approximately 28–32 percentage points across all test sequences (e.g., from 88.5% down to 56.3% on Seq-06). A similar drastic degradation is observed for Recall@1%. This clearly demonstrates that enforcing intra-modal consistency between multi-scale features is not merely an auxiliary task but a fundamental component for learning scale-invariant and discriminative descriptors. Without this supervision, the network fails to build a coherent hierarchical representation, resulting in poor retrieval accuracy.

Impact of the Weighting Factor λ: To pinpoint the optimal contribution of the Scale Consistency Loss, we conducted a fine-grained analysis with λ∈{0, 0.25, 0.5, 0.75, 1.0}. As shown in Table 6, the results exhibit a clear bell-shaped trend. Introducing the auxiliary loss with λ=0.25 yields significant improvements over the baseline, yet it falls short of the peak performance. The performance reaches its maximum at λ=0.5. However, further increasing λ beyond this point leads to progressive degradation. For instance, on Seq-05, Recall@1 drops from 89.4% (λ=0.5) to 80.1% (λ=0.75) and finally to 76.9% (λ=1.0). This confirms that λ=0.5 provides the optimal balance, effectively regularizing the feature hierarchy without interfering with the primary global contrastive learning objective.

### 4.8. Backbone Architecture Comparison

To validate our choice of Swin Transformer as the feature extraction backbone, we conducted a systematic comparison with three representative architectures under identical training settings:

**ResNet-50 + FPN:** While the Feature Pyramid Network provides multi-scale features, the CNN-based architecture captures strong local patterns but exhibits limited global context modeling capability, resulting in an average Recall@1 of 70.2%. **ViT-Small:** The Vision Transformer excels at capturing global dependencies through self-attention but lacks the hierarchical multi-scale structure inherent to our teacher–student paradigm. Its uniform patch-based design is less suitable for the spatially structured range/depth images, achieving 75.9% average recall. **ConvNeXt-Tiny:** This modernized CNN architecture balances local and global modeling, achieving 78.5% average recall. However, it still falls short of Swin Transformer’s performance due to its lack of explicit hierarchical attention mechanisms. **Swin Transformer (Ours):** Achieves the highest average Recall@1 of 86.6%, outperforming ConvNeXt by +8.1%, ViT by +10.7%, and ResNet + FPN by +16.4%. As shown in Table 7, these results validate that Swin Transformer is the optimal backbone choice for our multi-scale self-supervised cross-modal learning framework. The superior performance of the Swin Transformer over the standard ViT-Small can be attributed to its hierarchical architecture. While ViT processes images as uniform patches with a constant resolution, the Swin Transformer employs a shifted window mechanism to construct hierarchical feature maps. This structure allows our model to capture both fine-grained local geometric details (essential for depth-to-point cloud matching) and high-level semantic context, which aligns perfectly with our multi-scale self-distillation paradigm.

**Figure 4:**
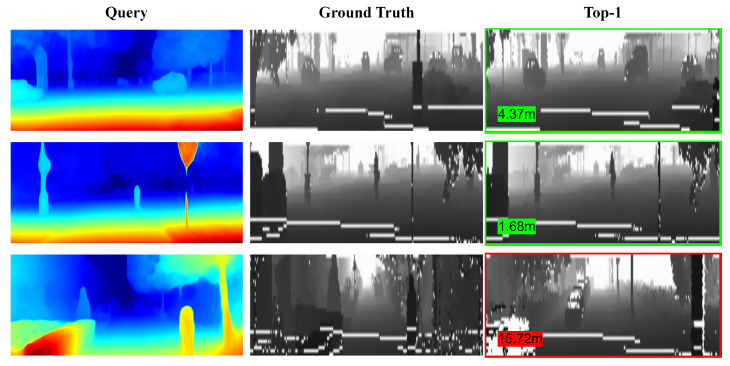
Qualitative retrieval results on the KITTI dataset. (**Left**): The query depth image. (**Middle**): The ground truth point cloud (projected as a range image). (**Right**): The Top-1 retrieved candidate. Green borders indicate successful matches (error < 10 m), while the red border highlights a failure case. The numbers denote the localization error in meters.

Qualitative retrieval results are visualized in Figure 4, which further confirms the effectiveness of our method.

### 4.9. Complexity and Runtime Analysis

All experiments were conducted on a workstation equipped with an Intel i7-13700K CPU (Intel, Santa Clara, CA, USA) and an NVIDIA GeForce RTX 4090 GPU (NVIDIA, Santa Clara, CA, USA). To validate the efficiency of the proposed MS2-CL, we analyze its model size and inference latency. The total number of parameters of our network is **29.6 M**. Note that the standard ResNet backbone already accounts for **26.9 M**, meaning our proposed cross-modal learning modules introduce only a marginal increase of **2.7 M** parameters, adhering to a lightweight design.

In terms of inference speed, our method is highly efficient. Excluding the offline depth estimation step, the feature extraction process takes an average of **5.0 ms** per frame. Although depth estimation is performed offline in our experiments for training efficiency, state-of-the-art lightweight monocular depth estimation networks can achieve inference latencies of approximately 25–30 ms on modern onboard GPUs. Consequently, the complete pipeline, including online depth estimation and retrieval, remains well within the real-time latency constraints required for autonomous driving systems. Candidates are sorted based on the magnitude of cosine similarity, enabling our Top-1 point cloud retrieval to only take **0.03 ms**. This results in a real-time performance suitable for onboard deployment.

## 5. Discussion

While our experimental results demonstrate the superior retrieval performance of MS2-CL on benchmark datasets, it is essential to contextualize these findings within real-world autonomous driving tasks. In this section, we discuss the practical implications of our image-to-point cloud place recognition framework, specifically focusing on global initialization, loop closure, and system redundancy.

### 5.1. Global Initialization in HD Maps

A critical challenge in autonomous driving is the “Kidnapped Robot Problem,” where a vehicle must determine its global pose within a map without any prior knowledge of its location. This typically occurs when a vehicle starts up in a GNSS-denied environment, such as an underground parking lot or an urban canyon. In this scenario, high-precision localizers often fail because they require a good initial pose guess to converge. Our MS2-CL serves as a robust **Global Initialization module**. By retrieving the most similar LiDAR submap from the pre-built 3D HD map using only the current camera view, our method provides a reliable coarse location estimate. This estimate acts as the initial seed for subsequent fine-grained 6-DoF pose estimation algorithms, significantly narrowing the search space and preventing local minima convergence.

### 5.2. Cross-Modal Loop Closure Detection

Long-term Simultaneous Localization and Mapping (SLAM) systems inevitably suffer from position drift over time. In a multi-sensor setup, our method functions as an effective **Loop Closure Detection** module. Consider a scenario where a mapping vehicle equipped with LiDAR has previously traversed a route, creating a geometric map. Later, a user vehicle equipped only with cameras revisits the same route. MS2-CL can identify this revisit by matching the current visual frame against the historical geometric map. By establishing a constraint between the current visual observation and the stored map, the backend optimization system (e.g., Pose Graph Optimization) can correct the accumulated drift, thereby maintaining the global consistency of the trajectory.

### 5.3. Safety Redundancy and Cost Efficiency

For Level 4 and Level 5 autonomous driving systems, sensor redundancy is a non-negotiable requirement for safety certification. In scenarios where primary localization sensors degrade—for example, if the GNSS signal is jammed or the on-board LiDAR fails due to mechanical issues—our vision-based retrieval against the 3D database provides a critical safety fallback layer. Furthermore, this cross-modal capability supports a cost-efficient fleet management model. It allows for a heterogeneous fleet structure: a small number of expensive, LiDAR-equipped vehicles can be responsible for maintaining the HD map, while a large fleet of mass-produced, camera-only vehicles can localize themselves within that map. This asymmetry significantly reduces the hardware cost for end-user vehicles while leveraging the high accuracy of LiDAR-based maps.

## 6. Conclusions

We proposed MS2-CL, a lightweight framework that significantly advances cross-modal place recognition. Distinct from methods focusing solely on cross-modal alignment, our approach introduces an intra-modal Scale Consistency Loss. This loss complements a global contrastive objective by ensuring features learned via a teacher–student paradigm are tightly aligned across multiple scales. This dual-supervision strategy leads to state-of-the-art results on the KITTI benchmark. Furthermore, our discussion on practical applications highlights the critical utility of MS2-CL in real-world autonomous driving tasks, specifically for global initialization in HD maps, cross-modal loop closure detection, and serving as a cost-effective redundancy layer for safety-critical systems. Finally, the model’s exceptional zero-shot performance on the out-of-distribution KITTI-360 dataset validates its strong generalization capabilities and potential for scalable deployment.

## Figures and Tables

**Table 1:** The Recall@1 and Recall@1% (%) of Image-to-Point Cloud Recognition on the KITTI dataset.

Method	Seq-00		Seq-02		Seq-05		Seq-06		Seq-08	
	@1	@1%	@1	@1%	@1	@1%	@1	@1%	@1	@1%
Baseline [28]	58.0	_	4.0	_	10.6	_	22.3	_	5.3	_
MIM-Points [35]	53.3	71.6	6.9	57.1	14.8	68.3	20.0	44.4	12.5	68.6
PSM-Points * [36]	58.0	72.3	8.2	53.6	16.0	71.1	32.1	50.0	14.7	75.0
LEA-Points * [37]	58.8	72.6	9.1	61.3	21.8	80.0	31.6	71.6	18.0	76.3
MIM-I2P-Rec [7]	74.3	88.6	46.3	89.7	49.9	89.1	41.9	81.5	42.1	88.4
PSM-I2P-Rec * [7]	81.7	97.4	47.6	92.9	63.8	94.1	52.1	88.5	53.5	92.1
LEA-I2P-Rec * [7]	92.6	99.7	77.0	98.4	83.4	98.8	55.5	91.5	69.4	96.4
(LC)^2^ [8]	31.4	84.5	23.1	67.4	40.5	88.6	39.8	71.5	38.4	87.3
LIP-Loc [30]	93.5	**100**	62.8	86.7	66.3	97.8	65.4	80.6	66.4	98.3
ModaLink [31]	98.0	**100**	70.5	97.7	**91.3**	99.3	87.4	**100**	84.4	99.9
MS2-CL (ours)	**98.4**	**100**	**81.7**	**99.8**	89.4	**99.8**	**88.5**	**100**	**86.8**	**100**

* indicates methods requiring stereo images as input. Bold values indicate the best performance in each column.

**Table 2:** Input modality comparison between MS2-CL and baseline methods. We report average Recall@1 (%) across all test sequences on the KITTI dataset. Methods marked with * require stereo image pairs as input.

Method	Input	Avg Recall@1 (%)	Hardware
PSM-I2P-Rec*	Stereo	59.8	2 cameras
LEA-I2P-Rec*	Stereo	75.8	2 cameras
LIP-Loc	Monocular	70.9	1 camera
ModaLink	Monocular	86.3	1 camera
**MS2-CL (Ours)**	**Monocular**	**88.9**	**1 camera**

**Table 3:** The Recall@**1** (%) comparison between MS2-CL and ModaLink against different distance thresholds on the KITTI dataset.

Sequence	Method	Threshold (m)					
		1	4	7	10	13	16
KITTI-02	ModaLink	59.3	60.1	64.3	70.5	71.7	72.9
	**MS2-CL**	**68.1**	**75.7**	**78.9**	**81.7**	**83.1**	**84.4**
KITTI-05	ModaLink	71.7	**82.2**	85.7	**91.3**	**92.5**	**92.7**
	**MS2-CL**	**72.0**	80.2	**86.1**	89.4	90.4	90.7
KITTI-06	ModaLink	66.2	73.1	80.9	87.4	87.7	88.2
	**MS2-CL**	**70.6**	**79.4**	**85.5**	**88.5**	**88.7**	**89.6**
KITTI-08	ModaLink	75.2	80.6	82.3	84.4	85.1	86.3
	**MS2-CL**	**76.3**	**82.3**	**83.6**	**86.8**	**87.8**	**88.9**

**Table 4:** Comparison of Image-to-Point Cloud Place Recognition performance on Sequence-0 of the KITTI-360 dataset.

Method	Accuracy (%)			
	Recall@1	Recall@5	Recall@10	Recall@20
AECMLoc [38]	46.2	66.0	72.5	78.2
Zero-shot LIP-Loc [30]	54.0	77.0	86.0	91.9
LIP-Loc [30]	68.6	86.6	92.7	96.6
Zero-shot MS2-CL(ours)	61.8	79.3	90.4	95.1
MS2-CL(ours)	**79.8**	**91.0**	**96.4**	**99.4**

AECMLoc requires fisheye images as input.

**Table 5:** Performance of Zero-shot MS2-CL without fine-tuning on the KITTI-360 dataset.

Sequence	0	3	4	5	6	7	9	10
Recall@1 (%)	61.8	62.4	59.7	66.5	57.5	60.3	64.9	58.2

**Table 6:** Ablation study on the weight of the Scale Consistency Loss (λ). We investigated a finer range of values (λ∈{0, 0.25, 0.5, 0.75, 1.0}) to identify the optimal balance. We report Recall@1 (%) and Recall@1% (%) on sequences from the KITTI dataset.

	Seq-02		Seq-05		Seq-06		Seq-08	
Value of λ	@1	@1%	@1	@1%	@1	@1%	@1	@1%
0.0 (Baseline)	53.6	78.1	60.8	81.5	56.3	79.3	59.2	80.4
0.25	55.1	80.3	61.2	81.8	57.8	80.1	59.9	81.5
**0.5 (Ours)**	**81.7**	**99.8**	**89.4**	**99.8**	**88.5**	**100**	**86.8**	**100**
0.75	74.5	95.5	80.1	95.9	77.5	93.4	73.4	95.2
1.0	73.1	94.7	76.9	95.8	69.2	91.5	71.2	93.1

**Table 7:** Ablation study on backbone architectures. All models are trained with the same multi-scale contrastive learning framework (λ=0.5). We report Recall@1 (%) and Recall@1% (%) on sequences from the KITTI dataset.

	Seq-02		Seq-05		Seq-06		Seq-08	
Backbone	@1	@1%	@1	@1%	@1	@1%	@1	@1%
ResNet-50 + FPN	68.3	95.2	72.1	96.8	70.8	94.5	69.5	95.1
ViT-Small	73.5	96.8	78.9	97.5	76.2	96.2	74.8	96.9
ConvNeXt-Tiny	75.8	97.5	81.3	98.2	79.4	97.8	77.6	97.3
**Swin-Tiny (Ours)**	**81.7**	**99.8**	**89.4**	**99.8**	**88.5**	**100**	**86.8**	**100**

## Data Availability

The code and pre-trained models are available at https://github.com/ml-bupt, accessed on 26 February 2026.
